# Influence of overnight orthokeratology on tear film and meibomian glands in myopic children: a prospective study

**DOI:** 10.1186/s12886-023-02883-8

**Published:** 2023-04-03

**Authors:** Jing Ruan, Yu Zhang, Yueguo Chen

**Affiliations:** Department of Ophthalmology, Beijing Key Laboratory of Restoration of Damaged Ocular Nerve, Peking University Third Hospital, 49 North Huayuan Road, Haidian District, Beijing, 100191 China

**Keywords:** Children, Cornea, Dry eye, Meibomian gland, Orthokeratology lenses

## Abstract

**Background:**

Orthokeratology lenses, which are worn overnight, are recommended for reducing myopia progression. They lie on the cornea and can influence the ocular surface by temporarily reshaping the corneal surface through a reverse geometry design. This study investigated the effect of overnight orthokeratology lenses on tear film stability and meibomian gland status in children aged 8–15 years.

**Methods:**

This prospective, self-controlled study included 33 children with monocular myopia who were prescribed orthokeratology lenses for at least one year. The experimental group (ortho-k group) comprised 33 myopic eyes. The control group comprised the emmetropic eyes of the same participants. Tear film stability and meibomian gland status were measured using a Keratograph 5M (Oculus, Wetzlar, Germany). Paired *t*-tests and Wilcoxon signed-rank tests were used to compare the data between the two groups.

**Results:**

At the one-year visit, the non-invasive first tear film break-up time (NIBUTf) values were 6.15 ± 2.56 s and 6.18 ± 2.61 s in the experimental and control groups, respectively. The lower tear meniscus height was 18.74 ± 0.05 μm and 18.65 ± 0.04 μm in these groups, respectively. No significant difference was observed in loss of meibomian glands or non-invasive average tear film break-up time between the experimental and control groups using Wilcoxon signed-rank tests.

**Conclusions:**

The stability of the tear film and meibomian gland status were not significantly affected by wearing orthokeratology lenses overnight, indicating that continuous use of orthokeratology lenses for 12 months has a minimal effect on the ocular surface. This finding can help guide the clinical management of tear film quality with respect to the use of orthokeratology contact lenses.

## Background

Myopia has emerged as a global health risk in the last few decades. The prevalence of myopia is reportedly to be 80–90% in adolescent aged 17–18 years in Southeast Asia [1], and nearly half of the global population suffers from ametropia, which causes blurred vision [2–4]. Severe myopia is associated with a series of vision-threatening ocular complications, such as glaucoma or myopic macular degeneration, which imposes a social burden [5, 6].

Traditional spectacle lenses do not help reduce the progression of myopia. Overnight use of orthokeratology (ortho-k) lenses is widely recommended by ophthalmologists and optometrists for reducing the progression of myopia, in particular, to prevent the occurrence of high myopia [4, 7]. Ortho-k lenses are inverse geometric lenses that help achieve good unaided vision; however, they cause redistribution of the anterior corneal epithelial and stromal tissues [8, 9]. Mousavi et al. [10] proved that ocular surface changes during the early stages of usage of the ortho-k lens, and structural changes in the cornea contribute to contact lens-associated discomfort. As ortho-k lenses lie directly on the corneal surface, their influence on the health of the ocular surface warrants investigation. Tear film stability and meibomian gland function are important indices of the ocular surface’s condition [3]. A previous study demonstrated changes in the ocular surface function in the first month of ortho-k lens wear. However, it gradually returned to normal [11]. Especially, if someone stops wearing the lenses at night, then, the eyes would eventually go back to their original shape and the refractive error would return. Another study suggested that the ortho-k lens has no effect on the ocular surface (only a reduction in central corneal sensitivity was observed) [8]. However, these studies were based on comparisons between two groups of patients, or between pre- and post-ortho-k lens treatment. Therefore, the findings might have been influenced by other factors, such as environment and lifestyle habits, which probably were unavoidable. Thus, their conclusions were controversial, and the outcomes were unclear.

Most ocular surface-related examinations are invasive, and the accuracy of the results is affected by many factors. Thus, some studies have used the Oculus Keratograph 5M (Oculus, Wetzlar, Germany) to obtain reliable results [9]. The Oculus integrated ocular surface analyzer (Keratograph 5M) provides a series of non-invasive examinations that can automatically measure the tear break-up time (BUT), amount of tear secretion, and number of meibomian glands, without requiring any staining.

This study aimed to determine whether ortho-k lenses change the ocular surface environment, and affect the function of meibomian glands in children. To exclude individual differences and external factors affecting the ocular surface, a unique method involving the same participants in the experimental and control groups was incorporated in the study. The tear film and meibomian gland were compared in children aged 8–15 years with monocular myopia, who had worn an ortho-k lens in one eye for at least 1 year, with those of the contralateral eye.

## Methods

### Participants

This prospective study included 33 patients with monocular myopia recruited from the Peking University Third Hospital (Beijing, China), from January 2018 to January 2021. Only one group of participants was included in the study; The patients in this group had one myopic eye and one emmetropic eye. The emmetropic eye was considered as the control group. The myopic eye of each participant received an overnight ortho-k lens, while the patient’s contralateral emmetropic (spherical equivalent refraction, < 0.50 D) eye did not receive an ortho-k lens from the beginning to the end of the study. The ortho-k and control groups included 33 myopic eyes and 33 emmetropic eyes, respectively. The inclusion and exclusion criteria are listed in Table 1.

**Table 1:** Inclusion and exclusion criteria

Inclusion Criteria	Exclusion Criteria
One myopic eye	Poor compliance with follow-up visits
Myopia ≤-6.00 D	Strabismus or amblyopia
Astigmatism ≤-1.50 D (with the rule)	Systemic disease or usage of drugs that affect the corneal endothelium
Overnight use of ortho-k lenses for at least 1 year	Stopped wearing lenses > 14 days per year
No prior use of contact lenses	Complications, such as keratitis, iridocyclitis, and continuous corneal epithelial damage, attributable to wearing the lens

Written informed consent was obtained from the parents or guardians of the participants before the commencement of the study. The study was approved by the ethics committee of Peking University Third Hospital in China and was conducted following the tenets of the Declaration of Helsinki.

### Examination

All participants underwent baseline comprehensive ophthalmological examinations, including measurement of uncorrected visual acuity, intraocular pressure (IOP, non-contact), cycloplegic refraction, best-corrected visual acuity (BCVA), and axial length. Slit-lamp examination, ocular fundus examination (Zeiss IOL Master V.5.02; Carl Zeiss Meditec, Jena, Germany), corneal endothelial microscopy, and corneal topography (Sirius; CSO, Florence, Italy) were also performed. Tear BUT, which reflects the quality and stability of the tear film, was measured under a slit lamp by the same ophthalmologist. To this end, fluorescein was applied and the cornea of the participant was evaluated under cobalt blue light; the examiner recorded the time in seconds from blinking to the first rupture point on the tear film.

### Lens selection

For the study patients, the first trial lens was selected based on the flat keratometry values and spherical equivalent refraction measured using corneal topography. Following tear film stabilization, the ophthalmologist observed the lens by staining with fluorescein, adjusted the trial lens until a satisfactory fit was observed, and used a spectacle lens for additional optical examination to obtain the final lens prescription for overnight ortho-k lenses. An ideal lens fit permitted appropriate lens movement (up and down ≤ 1 mm) during blinking with no obvious lens decentration.

In this study, three brands of ortho-k lenses were used: Lucid (Lucid, Bonghwa, Korea; BOSTON XO, DK = 100 × 10^11^ cm^2^/s), CRT (Paragon Vision Sciences, Mesa, AZ, USA; Paragon HDS 100, DK = 100 × 10^11^ cm^2^/s), and Euclid (Euclid Systems Corporation, Sterling, VA, USA; BOSTON EQUALENS, DK = 127 × 10^11^ cm^2^/s), which were used in 36.3, 39.4, and 24.3% of participants, respectively.

### Care and follow-up

All participants were required to wear the ortho-k lens every night. They were instructed to use appropriate care solutions for lens cleaning and disinfection every morning and night (Boston multi-action solution; Bausch + Lomb, Laval, Canada), and to remove the protein attached to the lens every 2 weeks (Menicon Progent A/B; Menicon, Nagoya, Japan) under parental assistance. Preservative-free sodium hyaluronate eye drops were used as lubricants when removing and wearing the ortho-k lenses. Participants attended follow-up examinations after the first night and subsequently one week, one month, and every three months following the commencement of lens wear (average of seven follow-up examinations).

No abnormalities were detected using slit-lamp microscopy during the follow-up period. Routine follow-up examinations were performed every 3 months, including BCVA measurement, slit-lamp microscopy, corneal topography, evaluation of the fit of the lens, and clearance inspection. The same technician conducted the 1-year examination for all patients using the Oculus Keratograph 5M. The parameters measured included the lower tear meniscus height, non-invasive first tear film BUT (NIBUTf), non-invasive average tear film BUT (NIBUTav), and degree of loss of the meibomian glands. The lower tear meniscus height was based on the position of the lacrimal river. A photograph was taken, and the lacrimal river height (middle area) at the lower eyelid was measured using a ruler provided by Oculus. The average result of three measurements was recorded. To determine the loss of the meibomian glands, examiners turned the upper and lower eyelids of participants over, used infrared light to photograph the position of the meibomian glands (enhanced contrast mode), and recorded the rate of loss of the meibomian glands as < 1/3, 1/3–2/3 or > 2/3.

The lighting system of the Keratograph 5M is mainly composed of diodes emitting red light within the safe range for human eyes; these have no noticeable effect on the tear film, and rarely induce reflective tears. The instrument can record videos of NIBUTf, NIBUTav, and changes in the tear film (lipid layer), and computes the average of the results of three tests as the final test outcome for both eyes. When the lower tear meniscus height and NIBUT were measured, a three-minute interval was observed between each inspection to permit sufficient blinking and rest of the inspector. A single inspector performed all the examination, who was also blinded to the participants wearing an Ortho-K lenses or not (single blindness).

### Statistical analysis

Statistical analysis was performed using IBM SPSS Statistics (Version 21.0. IBM Corp, Armonk, NY, USA). The normality of the samples was analyzed using the Shapiro–Wilk test. The paired sample t-test (normally distributed data) was used to compare the baseline data of the left and right eyes within the group. The results of LTMH and NIBUTf were normally distributed, so the Levene’s test of equality of variance was used. After a normal distribution was not confirmed, the Wilcoxon signed-rank test was used for NIBUTav, and the nonparametric test (Pearson’s chi-squared test) was used to compare the data between the two groups of LMG. Statistical significance was defined as *P* < 0.05.

## Results

Thirty-three participants (18 women and 15 men), with a mean age of 12.43 ± 2.27 (range, 8–15) years, were included in this study. All participants wore ortho-k lenses for nearly 8 h every night during the study period and underwent regular follow-up examinations. The average treatment time was 17.96 ± 4.14 months. No complications were recorded, and none of the patients dropped out during the study.

### Baseline data of the study participants

At baseline, the mean spherical equivalent of the ortho-k group was − 2.11 ± 0.97 D. No significant differences were observed in the baseline spherical equivalent refraction, astigmatism, BCVA, IOP, tear BUT, or flat and steep keratometry between the two groups (Table 2).

**Table 2:** Summary of the baseline data in the two groups

Eye	Ortho-k Group (n = 33)	Control Group (n = 33)	*P-*value
SER, D Astigmatism, D	-2.14 ± 0.97 -0.33 ± 0.43	0.26 ± 0.49 -0.39 ± 0.55	< 0.001 0.655
BCVA, LogMAR	1.0 ± 0.14	1.0 ± 0.15	0.088
IOP, mmHg	14.82 ± 2.63	14.63 ± 3.00	0.819
Flat keratometry, D Steep keratometry, D	42.89 ± 1.41 44.07 ± 1.67	42.83 ± 1.39 44.08 ± 1.59	0.883 0.989
Axial length, mm BUT, s	24.38 ± 0.77 7.26 ± 0.77	23.56 ± 0.72 7.00 ± 0.69	0.000 0.201

BCVA, best-corrected visual acuity; BUT, tear film break-up time; IOP, intraocular pressure; LogMAR, Logarithm of the Minimum Angle of Resolution; Ortho-k, orthokeratology; SER, spherical equivalent refraction.

### Comparison of tear film stability

There were no significant differences in the NIBUTf and NIBUTav between the two groups (Table 3).

**Table 3:** Comparison of the tear break-up time between the two groups

	Ortho-k Group	Control Group	*P-*value
NIBUTf (s)	6.15 ± 2.56	6.18 ± 2.61	0.968
NIBUTav (s)	6.85 ± 2.72	6.79 ± 3.04	0.900

NIBUTf, non-invasive first tear film break-up time; NIBUTav, non-invasive average tear film break-up time; Ortho-k, orthokeratology.

### Comparison of the loss of meibomian glands

At 1-year follow-up examination, it was observed that in the majority of the participants in both groups (90.9% and 93.9%), there was loss of only < 1/3 of the meibomian glands, with the number of participants in the other loss of meibomian gland (LMG) groups (1/3–2/3 and > 2/3) being insignificant. This indicates that there was no significant detrimental effect of ortho-k lenses on the meibomian glands (Table 4).

**Table 4:** Loss of meibomian glands in the two groups

	Ortho-k Group (n = 33)	Control Group (n = 33)	*P-*value
LMG, n (%)			0.642
< 1/3	30 (90.9%)	31 (93.9%)	
1/3–2/3	2 (9.1%)	3(6.1%)	
> 2/3	0 (0%)	0 (0%)	

LMG, loss of meibomian gland

### Comparison of the lower tear meniscus height

The lower tear meniscus height was 18.74 ± 0.05 and 18.65 ± 0.04 μm in the ortho-k and control groups, respectively. There was no significant difference between the two groups (t = -0.058, *P* = 0.954).

## Discussion

The efficacy of the ortho-k lens in myopia prevention and control has been confirmed by other studies; however, the safety of long-term overnight wear of ortho-k lenses in terms of corneal health and tear film quality required investigation. Nevertheless, monitoring symptoms of dryness of eye, and meibomian gland function is challenging, since they are influenced by multiple factors. Few studies have reported ocular surface changes in patients with monocular myopia using ortho-k lenses [2, 12, 13]. The results of the present study suggest that continuous use of ortho-k lenses for 12 months has a minimal effect on the ocular surface.

The Oculus Keratograph 5M is a more accurate tool for the measurement of tear film function than any other known device, as it helps control the variables in the ocular surface environment that induce dry eye symptoms [9, 14]. The Oculus Keratograph 5M could become a routine evaluation tool for the follow-up study of ortho-k lenses, and for monitoring the stability of the tear film, which is strongly related to lens-related discomfort [15].

Recently, some researchers who evaluated dryness of eyes and function of the meibomian glands reported that changes in the ortho-k lens parameters could have minimal effects on the corneal surface [13]. Kyung et al. examined 58 children in Seoul, South Korea, with myopia who were treated with ortho-k lenses [3]. In their 3-year study, the tear BUT showed no significant change in most children, which is similar to the findings of the present study. Furthermore, two participants had meibomian gland hypertrophy and deformation, suggesting that even small environmental changes can affect meibomian gland function in sensitive individuals.

A similar finding was also reported by Nieto-Bona et al. in 2018 [16]. In their 3-year clinical trial, 77 adult participants (23 without contact lenses, 26 wearing soft contact lenses, and 28 wearing ortho-k lenses) were evaluated using the TearLab Osmolarity System (TearLab, San Diego, CA, USA), to observe the effect of contact lens usage on the osmotic pressure of the tear film. They demonstrated that the tear film osmolarity of the contact lens wearers changed but returned to the initial level after discontinuation of ortho-k lenses for a few days. Thus, the effect of the ortho-k lens on the tear film was reversible and progression to dry eye was not observed. Furthermore, ortho-k lens wearers did not wear spectacles during the day, and experienced less discomfort.

Carracedo et al. reported that ortho-k lenses had no effect on conjunctival goblet cells [17]. The Schirmer test, tear BUT evaluation, and Ocular Surface Disease Index questionnaire survey were assessed in 22 participants following the use of ortho-k lenses for 1 month. The ortho-k lenses improved symptoms of dry eye discomfort following soft contact lens usage, and was considered as an alternative to soft contact lenses. Our results are consistent with those of recent studies.

In the present study, there was no significant difference in dry eye symptoms, height of the lacrimal river, and meibomian gland function between the eyes wearing ortho-k lenses and their contralateral eyes without lenses, which was consistent with the findings of Wang et al. [14]. Lam et al. also confirmed that ortho-k lenses did not affect meibomian gland function in a long-term study [18]. However, Berntsen et al. found irregularities in the eye surface morphology after wearing keratoplasty lenses in both eyes: wearing these lenses led to a decrease in the tear film and retinal imaging quality [19].

In a study by Tyagi et al. [20] on tear film surface quality, corneal hypoxia, and reduced tear film stability were observed with both soft contact lenses and rigid gas permeable lenses. Yang et al. demonstrated that short-term use of ortho-k lenses could reduce tear film stability and increase damage to the corneal epithelium [21]. These results differed from those of the present study. The discrepancy could be related to the design of the previous studies, in which two groups of individuals were studied: one group used an ortho-k lens and the other group did not. They compared tear film quality after a period of time, and concluded that wearing ortho-k lenses did not change the tear film quality. However, eliminating differences in the surrounding environment and eye habits is challenging. To our knowledge, only a few studies have compared the tear film quality of eyes in the same individuals before and after wearing ortho-k lenses [14].

To address the abovementioned shortcoming, this study included a binocular, self-control group comprising children with anisometropia, measured using the Oculus Keratograph 5M, to assess whether ortho-k lenses changed the ocular surface environment and affected meibomian gland function, resulting in eye dryness. The innovative inclusion criteria (i.e., myopic eyes wearing ortho-k lenses at night as the experimental group and non-myopic eyes of the same patients as the control group) reduced the impact of most of the confounding factors on the experimental results. There was no significant change in the tear film-breakup time and tear-river height after wearing the ortho-k lenses for 12 months. Such findings have not been reported in a similar study to date.

The limitations of this study were as follows. First, this study had a prospective design; however, data for Oculus (such as NIBUT and loss of meibomian glands) were not available before the intervention. Second, three different brands of ortho-k lenses were used in this study, and the differences in the material and lens design could have affected the results to some extent. Third, this study included only 33 participants, and a larger sample size is necessary to provide a more reliable conclusion. Finally, the mean follow-up period was 1.58 ± 0.37 years; therefore, a long-term study is warranted.

## Conclusions

Wearing ortho-k lenses did not significantly affect the tear film quality and the number of meibomian glands in children during the 12-month study. Overnight use of ortho-k lenses had a minimal effect on the ocular surface, while controlling the axial growth of the eye. The use of overnight ortho-k lenses could be considered safe because of its minimal effect on the ocular surface condition and meibomian gland function. Further long-term prospective studies are warranted to augment the results of this study.

## Data Availability

The datasets used and/or analysed during the current study are available from the corresponding author on reasonable request.

## Abbreviations

BCVA: Best-corrected visual acuity
BUT: Tear film break-up time
IOP: Intraocular pressure
LogMAR: Logarithm of the Minimum Angle of Resolution
ortho-k: Orthokeratology
LMG: Loss of meibomian gland
NIBUTf: Non-invasive first tear film break-up time
NIBUTav: Non-invasive average tear film break-up time
